# Key Aspects of Coronavirus Avian Infectious Bronchitis Virus

**DOI:** 10.3390/pathogens12050698

**Published:** 2023-05-11

**Authors:** Jing Zhao, Ye Zhao, Guozhong Zhang

**Affiliations:** 1 National Key Laboratory of Veterinary Public Health Security, College of Veterinary Medicine, China Agricultural University, Beijing 100193, China; 2 Key Laboratory of Animal Epidemiology of the Ministry of Agriculture, College of Veterinary Medicine, China Agricultural University, Beijing 100193, China

**Keywords:** coronavirus, avian infectious bronchitis virus, evolution, genetic diversity, antigenic diversity, vaccination

## Abstract

Infectious bronchitis virus (IBV) is an enveloped and positive-sense single-stranded RNA virus. IBV was the first coronavirus to be discovered and predominantly causes respiratory disease in commercial poultry worldwide. This review summarizes several important aspects of IBV, including epidemiology, genetic diversity, antigenic diversity, and multiple system disease caused by IBV as well as vaccination and antiviral strategies. Understanding these areas will provide insight into the mechanism of pathogenicity and immunoprotection of IBV and may improve prevention and control strategies for the disease.

## 1. Increasing Threats of Coronaviruses

Coronaviruses (CoVs) are enveloped, positive-sense, single-stranded RNA viruses belonging to the family *Coronaviridae* within the order *Nidovirales* and are widespread pathogens of significant potential threat to animals and humans [1,2,3]. The *Coronaviridae* are divided into alpha-, beta-, delta-, and gammacoronavirus genera (Table 1) and can infect a range of hosts, including humans, pigs, chickens, cows, horses, dogs, cats, turkeys, ducks, and many wild animals [1,4,5].

Among these CoVs, the porcine epidemic diarrhea virus and transmissible gastroenteritis virus of the Alphacoronavirus genus can impose a heavy disease burden on swine. Severe acute respiratory syndrome coronavirus (SARS-CoV), Middle East respiratory syndrome coronavirus (MERS-CoV), and SARS-CoV-2, which belong to the Betacoronavirus genus, have a significant mortality rate in humans. Porcine deltacoronavirus of the Deltacoronavirus genus infects piglets causing diarrhea, vomiting, and dehydration. Infectious bronchitis virus (IBV) of the Gammacoronavirus genus causes a highly contagious respiratory, renal, and genital disease in chickens known as avian infectious bronchitis (IB) [1,2,5,6].

IB is now a predominantly viral disease of commercial poultry worldwide that can affect chickens of all ages and types and has become a significant challenge for the poultry industry globally despite the extensive use of vaccination as a major control measure [7,8,9,10,11]. The major problem is that IBV constantly evolves via mutation and recombination to produce new variants or serotypes, and the protective immunity induced by one IBV serotype has poor cross-protection against infection by other serotypes.

## 2. Worldwide Distribution of Avian Infectious Bronchitis Virus

IBV was the first coronavirus to be discovered and was isolated from newly hatched chicks in the USA in 1931 [12]. The natural hosts of IBV are chicken, although it has also been isolated from other birds, including ducks, geese, pigeons, pheasants, peafowl, quail, parrots, penguins, and turkeys [8,13,14,15]. As shown in Table 1, IB was described before the 1990s in many countries following the first isolation of IBV. It is evident that IB has become a global disease with a wide distribution around the world [7,8]. Additionally, multiple genotypes or serotypes of IBV strains co-exist in many countries (Table 2) because of the rapid variation and spread and poor cross-protection among different mutants or serotypes.

In the USA, numerous IBV strains have been isolated since the 1930s, and major lineages belong to GI-9 (ArkDPI-like strains), GI-17 (DMV/1639-like strains), and GI-25 (CA1737-like strains) [12,16,17,18,53]. Currently, GI-17 (DMV/1639-like strains) is the major variant of IBV circulating in US commercial poultry; it was initially isolated in 2011 and began causing significant diseases in 2014/2015 [17]. In Latin America, lineages GI-1, GI-11, GI-16, and GI-23 are coexisting at present in Brazil, and the prevalent genotypes are GI-13 (793B-type) and GI-16 (Q1) in Chile [28,29,37]. In Africa, such as Egypt, Morocco, and Nigeria, the co-epidemic lineages include GI-1 (Mass-type) and GI-13 (793B-type); in addition to this, there are Egyptian IBV variants 1, 2 (GI-23) in Egypt, Moroccan isolates (GI-21) in Morocco, and QX-type (GI-19) strains in Nigeria [20,49,50,51]. In Australia and New Zealand, the co-epidemic lineages include GI-5 (N1/62) and GI-6 (Vic S), which caused mortalities of 40% to 100% between 1961 and 1976. Since 1988, N18/91 (GⅢ-3) and N1/03 (GⅤ) strains have been predominant in Australia, which mainly cause respiratory disease with lower mortality rates [32,53]. In Europe, the most common strains belong to lineages GI-13 (793B-type) and GI-19 (QX-type), followed by Mass-type (GI-1) strains in UK and Spain, D1466-like (GⅡ-1) and D181-like (GⅡ-2) strains in the Netherlands, and Italy 02 (GI-21) and D274 (GI-12) strains in Spain [8,26,27,38,39]. The GI-13 (793B-type) was detected most frequently in Europe, followed by GI-19 (QX-type) and GI-1 (Mass-type). In Asia, including Japan, Korea, India, Indonesia, Thailand, and China, QX-type (GI-19) strains are the most prevalent and exist in all six countries; 793B-type (GI-13) is the second popular lineage that exists in five countries except Korea; Mass-type (GI-1) strains are also isolated in India, Indonesia, Thailand, and China [22,23,24,34,40,41,42,43,44,45,46,48]. In addition, many other strains (lineages) are found in several countries, such as lineage GⅥ-1 (TC07-2) in Korea and China, Variant IBV (GI-24) in India, D85/06 (GI-15) in Korea, Taiwan-I (GI-7) in China, CU-92 (Novel variant) in Thailand, and Gray (GI-3), JP- I (GI-18) and JP-Ⅱ (GI-7) in Japan [8,22,34,44,46,48,53]. 

Especially in China, IBV was first described and isolated from chickens in the early 1980s. The virus has now spread to all poultry breeding areas. IB ranks first or second among the important infectious diseases of poultry in annual statistics in China and causes significant economic losses. Currently, at least four main IBV lineages, including QX-type (GI-19), Taiwan-I (GI-7), TC07-2 (GⅥ-1), and 793B-type (GI-13) coexist in chicken farms, which considerably increases the difficulty of prevention and control of the disease [11,43,44,54]. Among the four lineages, GI-19 (QX-type) lineage viruses are the most common strains with relatively higher pathogenicity, with a virus isolation rate of over 70%, whereas GⅥ-1 (TC07-2) lineage isolates have begun to significantly increase in prevalence in recent years, but have lower pathogenicity [8,9,43,44].

## 3. Genetic Diversity of Avian Infectious Bronchitis Virus

High rates of mutation, short generation times, and large population sizes drive the rapid evolution of RNA viruses, and consequently, most of the viral outbreaks over the last 100 years have been caused by RNA viruses [55]. Among the RNA viruses, positive-sense single-stranded RNA (+ssRNA) viruses such as IBV have the highest mutation rates [56,57]. IBV lacks a viral polymerase proofreading mechanism, and genetic mutation and recombination can continuously occur in the genome, especially in hypervariable regions of the S1 glycoprotein, which enables the continuous emergence of variants, genotypes, or serotypes [7,16,58,59].

**Figure 1:**
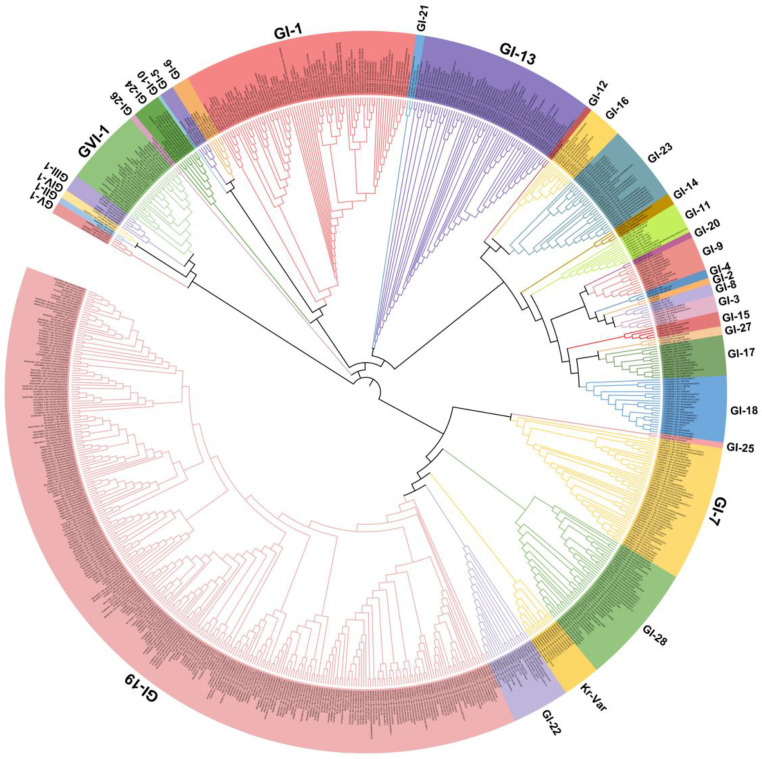
Phylogenetic trees based on the complete S1 glycoprotein gene of the infectious bronchitis virus (IBV). The tree was generated using the neighbor-joining method in MEGA 7 software package with 1000 bootstrap replications. The IBV genotypes were defined as described by Valastro et al. [53].

IBV strains are clustered into six genotypes (GⅠ–GⅥ) using the complete nucleotide sequences of the S1 gene, and together these contain 34 distinct viral lineages and several inter-lineage recombinants [53]. Of these genetic lineages, GⅠ-1, GⅠ-3, GⅠ-5, GⅠ-6, GⅠ-7, GⅠ-9, GⅠ-11, GⅠ-12, GⅠ-13, GⅠ-15, GⅠ-16, GⅠ-17, GⅠ-18, GⅠ-19, GⅠ-21, GⅠ-23, GⅠ-24, GⅠ-25, GⅡ-1, GⅡ-2,GⅢ-3, GⅤ, GⅥ-1, and several variants exist in many countries. The GⅠ-1 (Mass-type), GI-13 (793B-type), and GI-19 (QX-type) lineages are widely distributed in many countries, and especially QX-like viruses are now globally widespread (Figure 1). GI-7 (Taiwan-I) lineage strains have also been mainly isolated in China in recent years. Notably, live-attenuated vaccines of the Mass-type (e.g., strain H120), 793B-type (e.g., strain 4/91), Delmarva-type (e.g., DMV/1639 in the USA), and QX-type (e.g., QXL87 in China) are commonly used in many farms or countries [8,48,60,61,62,63]. Therefore, the possibility that several IBV strains are responsible for the re-isolation of the vaccine viruses cannot be excluded.

**Figure 2:**
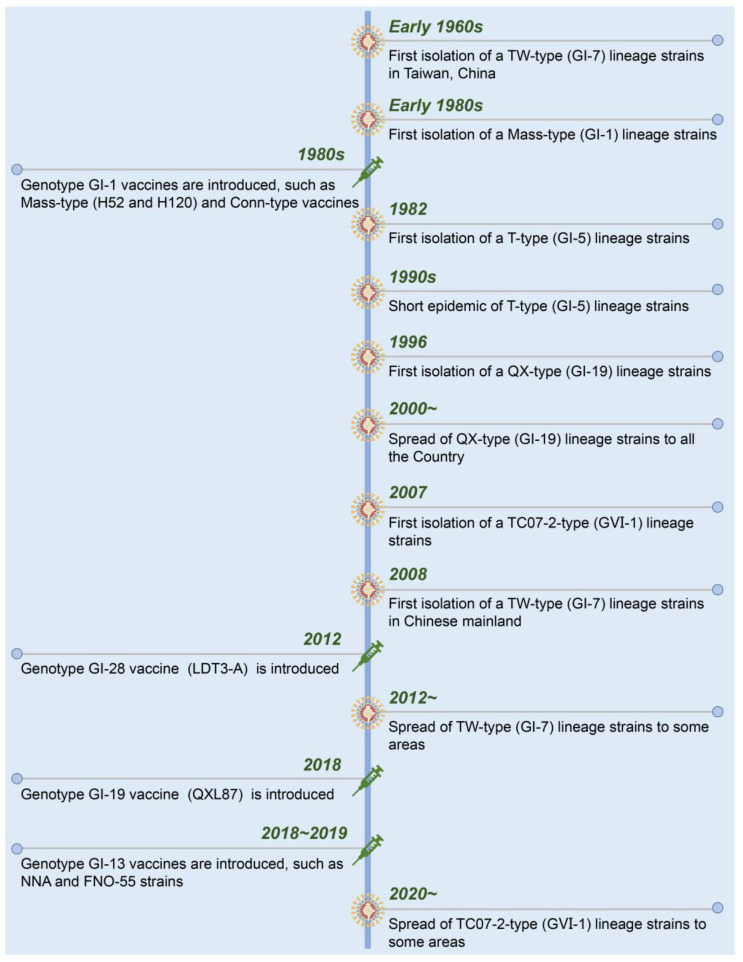
Chronological timeline showing the emergence and spread of the present IBV strains and introduction of live-attenuated vaccines in China.

A timeline with the date of isolation of each presenting IBV strain and used live-attenuated vaccines in China is shown in Figure 2. Suspected IB was first described in 1972 in Guangdong province, China [64]. Early in the 1980s, Mass-like IBV was isolated from Chinese chicken flocks that were genetically similar to that found in the USA. To control the diseases in chickens, the inactivated and live-attenuated vaccines of Mass (H52 and H120) and Conn serotypes were used. The IBV strains from T-type (GI-5), QX-type (GI-19), TC07-2-type (GⅥ-1), and TW-type (GI-7) were successively isolated from the vaccinated chicken flocks [65,66,67]. Currently, GI-19 (QX-type) and GⅥ-1 (TC07-2-type) lineage strains have become the dominant strains of IBV in China [68,69,70].

## 4. Antigenic Diversity of Avian Infectious Bronchitis Virus

New IBV variants continuously emerge through genetic recombination and mutation, and several exhibit clear antigenicity differences revealed by cross-neutralization tests or monoclonal antibody analysis. The GI-19 lineage (QX-type) strains, for instance, are prevalent in many parts of the world, and are distinct from all other known IBV strains, indicating that these strains belong to different serotypes [71,72,73]. Antigenic variation of IBV is mainly associated with the spike protein because this is a major inducer of neutralizing antibodies and immunoprotection [7,16,52,69]. Other proteins, including structural or nonstructural proteins and accessory proteins, contribute to and influence antigenic differences [7,59]. Thus, antigenic variation of IBV is caused by various factors. 

Currently, IBV exists as a serotype score worldwide, and most of these have poor cross-protection with each other [7,74,75,76,77]. This means that vaccination with a particular serotype of IBV may not provide protection against other serotypes. As a result of the antigenic variation, the disease presents a continuing coexistence of multiple serotyping strains, meaning that the production of new IB vaccines that better match the prevalent virus strains will likely continue for a long time. To address the major challenge of IBV antigenic diversity, researchers have focused on understanding the genetic mechanisms underlying IBV evolution, developing new vaccines with cross-protection, improving biosecurity measures to prevent viral spread, and so on.

## 5. Multiple System Disease Caused by Avian Infectious Bronchitis Virus

Most IBVs can infect the respiratory, renal, and reproductive systems of chickens. Following the initial infection in the respiratory tract with clinical signs (sneezing, gasping, coughing, tracheal rales, nasal discharge, and dyspnea), IBV is disseminated to other tissues by viremia [75]. One possible mechanism is that some strains could infect blood monocytes, and therefore facilitate the dissemination of IBV to visceral organs [78,79]. IBV dissemination beyond the respiratory tract may involve the lymphatic system and infected macrophages as with several other viruses [80,81]. Compared with other IBV strains, the nephropathogenic strains have received increased attention because of their higher virulence in young birds [42]. The kidneys of nephropathogenic IBV-infected chickens are pale, discolored, and enlarged with obvious urate deposition on post-mortem examination [79,82,83]. IBV infections in young chickens are common between the ages of 1 to 30 days and can cause reproductive tract defects [84,85,86]. The infected chickens develop cystic oviducts, leading to false layer syndrome with distinctly low peak egg production [86,87,88,89]. Infection with IBV in laying hens can negatively influence egg production with poor-quality eggs [90,91,92]. Several IBV strains can infect the nervous system of chickens [93]. These strains contain an extra furin cleavage site upstream of the fusion peptide (S2’ site) enabling them to infect monocytes and neuron cells, thus leading to viremia or encephalitis in chickens [78,93].

**Figure 3:**
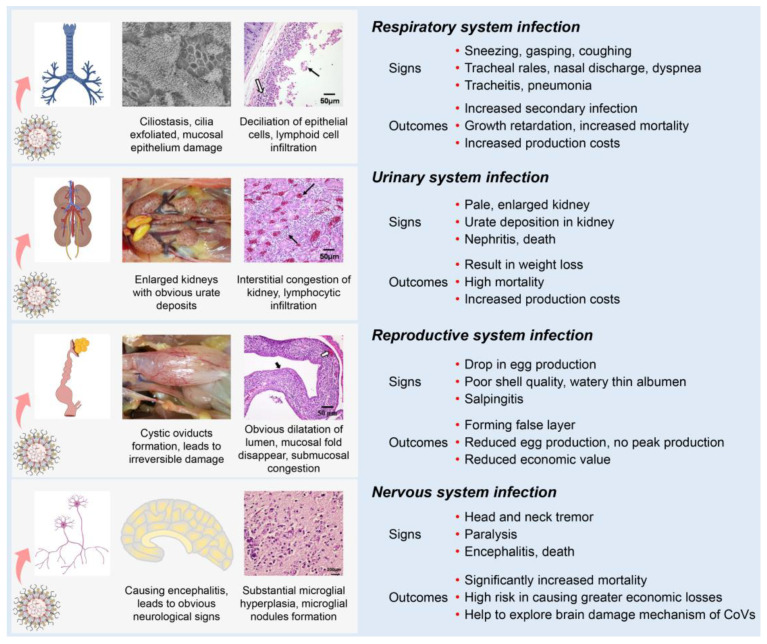
Schematic diagram showing clinical manifestations of IBV infection. Clinical signs and main outcomes are summarized, showing the effects of the virus on different body systems.

Although differences in clinical and pathological outcomes in chickens depend on the infecting IBV strain, many IBV strains can simultaneously infect multiple physiological systems in chickens, which is termed “multiple symptoms with one virus” (Figure 3). First, IBVs can infect the respiratory system of chickens, causing damage to the integrity of the respiratory mucosa, which could increase the susceptibility to secondary viral and bacterial infections [71,94,95,96]. Second, IBVs can infect the renal system, leading to increased mortality [71,82,95,97,98]. Third, IBVs can infect the reproductive system of chickens, reducing egg production with poor shell quality or with false layers [18,85,86,95,99]. Fourth, IBVs can infect the nervous system of chickens, causing significantly increased mortality [78,93]. Studies of IBV and host interactions could help to understand the tropism of IBV in various body systems and act as a model to contribute an invaluable resource for studying other pathogenic CoV diseases.

## 6. Prevention and Control of Avian Infectious Bronchitis Virus

In modern poultry farms, an effective management system and biosecurity measures are of primary importance to control infectious diseases. This premise is based entirely on the correct understanding of the factors influencing viral spread, as with the avian influenza virus [100,101,102]. Therefore, a more comprehensive analysis of the epidemiological aspects behind the spread of IBV, especially the spreading determinants, is critical and urgently required [103,104].

Despite drawbacks, including severe reactions to vaccination at the day of age, the likelihood of viral recombination, and frequent vaccine replacement caused by viral mutation, vaccination remains the most effective control measure for IB [105,106,107,108,109]. Efficient and properly performed vaccination may reduce the emergence of clinical signs, infectious pressure, and viral population size [105].

Currently, almost all commercial chicken flocks are vaccinated against IBV. The most effective IB vaccine is the live-attenuated vaccine, and H120 is one of the excellent vaccine strains widely used in the world. The vaccination protocol for IBV can vary depending on the vaccine used and the specific conditions of the poultry operation. In general, chicks are vaccinated at one day old or at hatchery, and then again several times throughout their life to maintain immunity. Booster vaccinations may be given at 7–10 days of age, at 3–4 weeks of age, and then every 5–6 weeks thereafter. Because of the many existing variants worldwide, the concept of protectotype has been increasingly accepted for controlling IB. One of the most commonly applied protectotype vaccination protocols against IB in the EU is the simultaneous or alternate use of Ma5 and 4/91 vaccine strains to provide protection against homologous or heterologous IBV strains [110]. In China, the H120 vaccine combined with an endemic strain, such as QXL87 (QX-like, GI-19), FNO-55 (4/91-like, GI-13), or LDT3-A (YN-like, GI-28), is commonly used to control IB.

Different vaccine platforms have been designed to develop effective vaccines against IBV. However, new vaccine techniques are rarely used because of the relatively complex immune protection mechanism and are currently only in the laboratory research stage [63,111,112,113,114,115,116,117]. Consequently, both inactivated and live-attenuated IBV vaccines are widely available worldwide [118,119,120,121].

## 7. Concluding Remarks and Future Directions

IB is a highly contagious viral disease caused by coronavirus IBV that produces severe economic losses in the poultry industry worldwide. Despite immunization and biosecurity measures, IB still frequently occurs in commercial chicken flocks because of the constant emergence of new IBV variants as well as poor cross-protection against them with the existing vaccines currently in use. Therefore, similarly to the threat of SARS-CoV-2 to human health, IB continuously threatens the healthy development of the poultry industry.

As with other CoVs, IBV undergoes rapid evolution, producing a growing number of genotypes and serotypes due to high rates of mutation, viral recombination, and host selection pressure. IBVs have a high genetic and antigenic diversity, whereas our knowledge of the ecology and evolution of IBVs remains very limited.

IBV infection can induce obvious respiratory and reproductive symptoms as well as increased mortality in chickens, especially when secondary infections of bacteria or viruses occur. “Multiple symptoms with one virus” caused by IBV affects chickens of various types and ages globally and most likely will continue to be a serious threat to the global poultry industry.

To prevent and control IB, the most effective measure at present remains vaccination despite current challenges. Clarifying the pathogenesis of the virus and optimizing the vaccination strategy using specific local strains will help to address continuing concerns over the efficacy of existing IB vaccines.

## Figures and Tables

**Table 1:** Classification of coronaviruses by the International Committee on Taxonomy of Viruses, and the major virus species belonging to the Coronaviridae family that threaten poultry and other livestock and pets or humans.

Order/Suborder	Family/Subfamily	Genus/Subgenus	Notable Virus Species	Main Host
*Nidovirales* *Cornidovirineae*	*Coronaviridae* *Orthocoronavirinae*	*Alphacoronavirus*		
		*Duvinacovirus*	Human coronavirus 229E (HCoV-229E)	Human
		*Pedacovirus*	Porcine epidemic diarrhea virus (PEDV)	Pig
		*Rhinacovirus*	Swine acute diarrhea syndrome coronavirus (SADS-CoV)	Pig
		*Setracovirus*	Human coronavirus NL63 (HCoV-NL63)	Human
			Alphacoronavirus 1	
			Transmissible gastroenteritis virus (TGEV)	Pig
			Feline infectious peritonitis virus (FIPV)	Cat
			Canine coronavirus (CCoV)	Dog
		*Betacoronavirus*		
		*Embecovirus*	Betacoronavirus 1	
			Human coronavirus OC43 (HCoV-OC43)	Human
			Bovine coronavirus (BCoV)	Cow
			Equine coronavirus (ECoV)	Horse
			Human coronavirus HKU1 (HCoV-HKU1)	Human
		*Merbecovirus*	Middle East respiratory syndrome-related coronavirus (MERS-CoV)	Human
		*Sarbecovirus*	Severe acute respiratory syndrome-related coronavirus	
			Severe acute respiratory syndrome-related coronavirus (SARS-CoV)	Human
			Severe acute respiratory syndrome-related coronavirus 2 (SARS-CoV-2)	Human
		*Deltacoronavirus*		
		*Buldecovirus*	Porcine deltacoronavirus (PDCoV)	Pig
		*Gammacoronavirus*		
		*Brangacovirus*	Goose coronavirus CB17 (GCoV-CB17)	Goose
		*Igacovirus*	Avian coronavirus	
			Infectious bronchitis virus (IBV)	Chicken
			Turkey coronavirus (TCoV)	Turkey
			Duck coronavirus 2714 (DCoV-2714)	Duck

**Table 2:** The first described outbreak and current situation of IB in several countries.

Year ^a^	Country	Host	Coexisting Strains/Genotype ^b^	Refs
1931	USA	Chicken	DMV/1639 (GI-17) CA1737 (GI-25) Mass-type (GI-1)	[12,16,17,18]
1954	Egypt	Chicken	EGY-var 1, 2 (GI-23) 793B-type (GI-13)	[19,20]
1954	Japan	Chicken	JP- I (GI-18) JP-Ⅱ (GI-7) JP-Ⅲ (GI-19, QX-type) JP-Ⅳ (GVI-1) 793B-type (GI-13) Mass-type (GI-1)	[21,22,23,24]
1956	Netherlands	Chicken	D1466 (GⅡ-1) D181 (GⅡ-2) QX-type (GI-19) 793B-type (GI-13)	[25,26,27]
1957	Brazil	Chicken	Mass-type (GI-1) SB2805 (GI-23) GI-11 GI-16	[28,29]
1960s	UK	Chicken	793B-type (GI-13) QX-type (GI-19) Mass-type (GI-1)	[8,30]
1962	Australia	Chicken	N1/62 (GI-5) Vic S (GI-6) N18/91 (GⅢ-3) N1/03 (GⅤ)	[31,32]
1962	Thailand	Chicken	QX-type (GI-19) Mass-type (GI-1) 793B-type (GI-13) CU-92 (Novel variant)	[33,34]
1967	New Zealand	Chicken	N1/62 (GI-5) Vic S (GI-6)	[8,35]
1969	Chile	Chicken	793B-type (GI-13) Q1 (GI-16)	[36,37]
Early 1970s	Spain	Chicken	QX-type (GI-19) 793B-type (GI-13) Mass-type (GI-1) Italy 02 (GI-21) D274 (GI-12)	[38,39]
1977	Indonesia	Chicken	QX-type (GI-19) 793B-type (GI-13) Mass-type (GI-1)	[40,41]
Early 1980s	China	Chicken	QX-type (GI-19) Taiwan-I (GI-7) TC07-2 (GⅥ-1) 793B-type (GI-13) Mass-type (GI-1)	[42,43,44]
1984	India	Chicken	Mass-type (GI-1) 793B-type (GI-13) Variant IBV(GI-24)	[45,46]
1986	Korea	Chicken	QX-type (GI-19) TC07-2 (GⅥ-1) D85/06 (GI-15)	[47,48]
1986	Morocco	Chicken	Moroccan isolates (GI-21) 793B-type (GI-13) Mass-type (GI-1)	[49,50]
1990	Nigeria	Chicken	Mass-type (GI-1) 793B-type (GI-13) Q1 (GI-16) QX-type (GI-19)	[51,52]

^a^ Time when the disease was first described. ^b^ In parentheses: genotype according to Valastro et al. [53].

## Data Availability

Not applicable.

## References

[1] Miranda C., Silva V., Igrejas G., Poeta P. (2021). Genomic evolution of the human and animal coronavirus diseases. Mol. Biol. Rep..

[2] Cui J., Li F., Shi Z.L. (2019). Origin and evolution of pathogenic coronaviruses. Nat. Rev. Microbiol..

[3] Kung Y.A., Lee K.M., Chiang H.J., Huang S.Y., Wu C.J., Shih S.R. (2022). Molecular virology of SARS-CoV-2 and related coronaviruses. Microbiol. Mol. Biol. Rev..

[4] Thakor J.C., Dinesh M., Manikandan R., Bindu S., Sahoo M., Sahoo D., Dhawan M., Pandey M.K., Tiwari R., Emran T.B. (2022). Swine coronaviruses (SCoVs) and their emerging threats to swine population, inter-species transmission, exploring the susceptibility of pigs for SARS-CoV-2 and zoonotic concerns. Vet. Q..

[5] Ruiz-Aravena M., McKee C., Gamble A., Lunn T., Morris A., Snedden C.E., Yinda C.K., Port J.R., Buchholz D.W., Yeo Y.Y. (2022). Ecology, evolution and spillover of coronaviruses from bats. Nat. Rev. Microbiol..

[6] Kesheh M.M., Hosseini P., Soltani S., Zandi M. (2022). An overview on the seven pathogenic human coronaviruses. Rev. Med. Virol..

[7] Cavanagh D. (2007). Coronavirus avian infectious bronchitis virus. Vet. Res..

[8] Bande F., Arshad S.S., Omar A.R., Hair-Bejo M., Mahmuda A., Nair V. (2017). Global distributions and strain diversity of avian infectious bronchitis virus: A review. Anim. Health Res. Rev..

[9] Zhao Y., Cheng J.L., Liu X.Y., Zhao J., Hu Y.X., Zhang G.Z. (2015). Safety and efficacy of an attenuated Chinese QX-like infectious bronchitis virus strain as a candidate vaccine. Vet. Microbiol..

[10] Jordan B. (2017). Vaccination against infectious bronchitis virus: A continuous challenge. Vet. Microbiol..

[11] Li Y.T., Chen T.C., Lin S.Y., Mase M., Murakami S., Horimoto T., Chen H.W. (2020). Emerging lethal infectious bronchitis coronavirus variants with multiorgan tropism. Transbound. Emerg. Dis..

[12] Schalk A.F., Hawin M.C. (1931). An apparently new respiratory disease in baby chicks. J. Am. Vet. Med. Assoc..

[13] Cavanagh D., Mawditt K., Welchman D.D.B., Britton P., Gough R.E. (2002). Coronaviruses from pheasants (*Phasianus colchicus*) are genetically closely related to coronaviruses of domestic fowl (infectious bronchitis virus) and turkeys. Avian Pathol..

[14] Chen G.Q., Zhuang Q.Y., Wang K.C., Liu S., Shao J.Z., Jiang W.M., Hou G.Y., Li J.P., Yu J.M., Li Y.P. (2013). Identification and survey of a novel avian coronavirus in ducks. PLoS ONE.

[15] Miłek J., Blicharz-Domańska K. (2018). Coronaviruses in avian species-review with focus on epidemiology and diagnosis in wild birds. J. Vet. Res..

[16] Ali A., Ojkic D., Elshafiee E.A., Shany S., El-Safty M.M., Shalaby A.A., Abdul-Careem M.F. (2022). Genotyping and in silico analysis of Delmarva (DMV/1639) infectious bronchitis virus (IBV) spike 1 (S1) glycoprotein. Genes.

[17] Jackwood M.W., Jordan B.J. (2021). Molecular evolution of infectious bronchitis virus and the emergence of variant viruses circulating in the United States. Avian Dis..

[18] Mueller Slay A., Franca M., Jackwood M., Jordan B. (2022). Infection with IBV DMV/1639 at a young age leads to increased incidence of cystic oviduct formation associated with false layer syndrome. Viruses.

[19] Ahmed H.N. (1954). Incidence and Treatment of Some Infectious Viral Respiratory Diseases of Poultry in Egypt. Ph.D. Thesis.

[20] Moharam I., Sultan H., Hassan K., Ibrahim M., Shany S., Shehata A.A., Abo-ElKhair M., Pfaff F., Höper D., El Kady M. (2020). Emerging infectious bronchitis virus (IBV) in Egypt: Evidence for an evolutionary advantage of a new S1 variant with a unique gene 3ab constellation. Infect. Genet. Evol..

[21] Nakamura J., Kuba N., Kawakubo A. (1954). A virus isolated from infectious bronchitis-like diseases of chickens. Jpn. J. Vet. Sci..

[22] Mase M., Hiramatsu K., Watanabe S., Iseki H. (2022). Genetic analysis of the complete S1 gene in Japanese infectious bronchitis virus strains. Viruses.

[23] Nakanishi M., Soma J., Takahashi S., Matsune K., Ono M., Oosumi T. (2022). Detection and isolation of QX-like infectious bronchitis virus in Japan. J. Vet. Med. Sci..

[24] Saito H., Nakagawa K., Kitamura Y., Kuwata K., Tanaka E. (2022). Molecular survey of infectious bronchitis virus on poultry farms in Gifu Prefecture, Japan from 2021 to 2022 by RT-PCR with an enhanced level of detection sensitivity for the S1 gene. J. Vet. Med. Sci..

[25] Kusters J.G., Niesters H.G., Bleumink-Pluym N.M., Davelaar F.G., Horzinek M.C., Van der Zeijst B.A. (1987). Molecular epidemiology of infectious bronchitis virus in The Netherlands. J. Gen. Virol..

[26] Sjaak de Wit J.J., Ter Veen C., Koopman H.C.R. (2020). Effect of IBV D1466 on egg production and egg quality and the effect of heterologous priming to increase the efficacy of an inactivated IBV vaccine. Avian Pathol..

[27] Molenaar R.J., Dijkman R., de Wit J.J. (2020). Characterization of infectious bronchitis virus D181, a new serotype (GII-2). Avian Pathol..

[28] Fraga A.P., Gräf T., Pereira C.S., Ikuta N., Fonseca A.S.K., Lunge V.R. (2018). Phylodynamic analysis and molecular diversity of the avian infectious bronchitis virus of chickens in Brazil. Infect. Genet. Evol..

[29] Ikuta N., Fonseca A.S.K., Fernando F.S., Filho T.F., Martins N.R.D.S., Lunge V.R. (2022). Emergence and molecular characterization of the avian infectious bronchitis virus GI-23 in commercial broiler farms from South America. Transbound. Emerg. Dis..

[30] Cavanagh D., Davis P.J. (1992). Sequence analysis of strains of avian infectious bronchitis coronavirus isolated during the 1960s in the U.K. Arch. Virol..

[31] Cumming R.B. (1962). The aetiology of “uraemia” of chickens. Aust. Vet. J..

[32] Quinteros J.A., Ignjatovic J., Chousalkar K.K., Noormohammadi A.H., Browning G.F. (2021). Infectious bronchitis virus in Australia: A model of coronavirus evolution—A review. Avian Pathol..

[33] Chindavanig P. (1962). Studies on the attenuation of infectious bronchitis virus. J. Thai. Vet. Med. Assoc..

[34] Munyahongse S., Pohuang T., Nonthabenjawan N., Sasipreeyajan J., Thontiravong A. (2020). Genetic characterization of infectious bronchitis viruses in Thailand, 2014–2016: Identification of a novel recombinant variant. Poult. Sci..

[35] Pohl R.M. (1967). Infectious bronchitis in chickens. New Zeal. Vet. J..

[36] Cubillos A., Ulloa J., Cubillos V., Cook J.K. (1991). Characterisation of strains of infectious bronchitis virus isolated in Chile. Avian Pathol..

[37] Guzmán M., Sáenz L., Hidalgo H. (2019). Molecular and antigenic characterization of GI-13 and GI-16 avian infectious bronchitis virus isolated in Chile from 2009 to 2017 regarding 4/91 vaccine introduction. Animals.

[38] Dolz R., Pujols J., Ordóñez G., Porta R., Majó N. (2008). Molecular epidemiology and evolution of avian infectious bronchitis virus in Spain over a fourteen-year period. Virology.

[39] Cortés V., Sevilla-Navarro S., García C., Marín C., Catalá-Gregori P. (2022). Seroprevalence and prevalence of infectious bronchitis virus in broilers, laying hens and broiler breeders in Spain. Poult. Sci..

[40] Setiawaty R., Soejoedono R.D., Poetri O.N. (2019). Genetic characterization of S1 gene of infectious bronchitis virus isolated from commercial poultry flocks in West Java, Indonesia. Vet. World.

[41] Wibowo M.H., Ginting T.E., Asmara W. (2019). Molecular characterization of pathogenic 4/91-like and QX-like infectious bronchitis virus infecting commercial poultry farms in Indonesia. Vet. World.

[42] Feng J., Hu Y., Ma Z., Yu Q., Zhao J., Liu X., Zhang G. (2012). Virulent avian infectious bronchitis virus, People’s Republic of China. Emerg. Infect. Dis..

[43] Fan W., Chen J., Zhang Y., Deng Q., Wei L., Zhao C., Lv D., Lin L., Zhang B., Wei T. (2022). Phylogenetic and spatiotemporal analyses of the complete genome sequences of avian coronavirus infectious bronchitis virus in China during 1985–2020: Revealing coexistence of multiple transmission chains and the origin of LX4-type virus. Front. Microbiol..

[44] Wang C.Y., Luo Z.B., Shao G.Q., Hou B. (2022). Genetic and pathogenic characteristics of a novel infectious bronchitis virus strain in genogroup VI (CK/CH/FJ/202005). Vet. Microbiol..

[45] Bayry J., Goudar M.S., Nighot P.K., Kshirsagar S.G., Ladman B.S., Gelb J., Ghalsasi G.R., Kolte G.N. (2005). Emergence of a nephropathogenic avian infectious bronchitis virus with a novel genotype in India. J. Clin. Microbiol..

[46] Raja A., Dhinakar Raj G., Kumanan K. (2020). Emergence of variant avian infectious bronchitis virus in India. Iran. J. Vet. Res..

[47] Rhee Y.O., Kim J.H., Mo I.P., Choi S.H., Namgoong S. (1986). Outbreaks of infectious bronchitis in Korea. Korean J. Vet. Res..

[48] Jung J.S., Lee R., Yoon S.I., Lee G.S., Sung H.W., Kwon H.M., Park J. (2022). Genetic and immunological characterization of commercial infectious bronchitis virus vaccines used in Korea. Arch. Virol..

[49] El-Houadfi M., Jones R.C., Cook J.K., Ambali A.G. (1986). The isolation and characterisation of six avian infectious bronchitis viruses isolated in Morocco. Avian Pathol..

[50] Fellahi S., El Harrak M., Khayi S., Guerin J.L., Kuhn J.H., El Houadfi M., Ennaji M.M., Ducatez M. (2017). Phylogenetic analysis of avian infectious bronchitis virus isolates from Morocco: A retrospective study (1983 to 2014). Virol. Sin..

[51] Shittu I., Gado D.A., Meseko C.A., Nyam D.C., Olawuyi K.A., Moses G.D., Chinyere C.N., Joannis T.M. (2019). Occurrence of infectious bronchitis in layer birds in Plateau state, north central Nigeria. Open Vet. J..

[52] Ekiri A.B., Armson B., Adebowale K., Endacott I., Galipo E., Alafiatayo R., Horton D.L., Ogwuche A., Bankole O.N., Galal H.M. (2021). Evaluating disease threats to sustainable poultry production in Africa: Newcastle disease, infectious bursal disease, and avian infectious bronchitis in commercial poultry flocks in Kano and Oyo States, Nigeria. Front. Vet. Sci..

[53] Valastro V., Holmes E.C., Britton P., Fusaro A., Jackwood M.W., Cattoli G., Monne I. (2016). S1 gene-based phylogeny of infectious bronchitis virus: An attempt to harmonize virus classification. Infect. Genet. Evol..

[54] Zhang X., Guo M., Zhao J., Wu Y. (2021). Avian infectious bronchitis in China: Epidemiology, vaccination, and control. Avian Dis..

[55] Grellet E., L’Hôte I., Goulet A., Imbert I. (2022). Replication of the coronavirus genome: A paradox among positive-strand RNA viruses. J. Biol. Chem..

[56] Echeverría N., Moratorio G., Cristina J., Moreno P. (2015). Hepatitis C virus genetic variability and evolution. World J. Hepatol..

[57] Thébaud G., Chadoeuf J., Morelli M.J., McCauley J.W., Haydon D.T. (2010). The relationship between mutation frequency and replication strategy in positive-sense single-stranded RNA viruses. Proc. Biol. Sci..

[58] Webb I., Keep S., Littolff K., Stuart J., Freimanis G., Britton P., Davidson A.D., Maier H.J., Bickerton E. (2022). The genetic stability, replication kinetics and cytopathogenicity of recombinant avian coronaviruses with a T16A or an A26F mutation within the E protein is cell-type dependent. Viruses.

[59] Kumar S., Thambiraja T.S., Karuppanan K., Subramaniam G. (2022). Omicron and Delta variant of SARS-CoV-2: A comparative computational study of spike protein. J. Med. Virol..

[60] Tizard I.R. (2020). Vaccination against coronaviruses in domestic animals. Vaccine.

[61] Abozeid H.H., Naguib M.M. (2020). Infectious bronchitis virus in Egypt: Genetic diversity and vaccination strategies. Vet. Sci..

[62] Hassan M.S.H., Buharideen S.M., Ali A., Najimudeen S.M., Goldsmith D., Coffin C.S., Cork S.C., van der Meer F., Abdul-Careem M.F. (2022). Efficacy of commercial infectious bronchitis vaccines against Canadian Delmarva (DMV/1639) infectious bronchitis virus infection in layers. Vaccines.

[63] Qin Y., Teng Q., Feng D., Pei Y., Zhao Y., Zhang G. (2022). Development of a nanoparticle multiepitope DNA vaccine against virulent infectious bronchitis virus challenge. J. Immunol..

[64] Xin C.A., Chen T.J. (1982). The research of chicken infectious bronchitis-I. Isolation and identification of chicken infectious bronchitis virus in Guangzhou. J. S. China Agric. Coll..

[65] Liu S., Wang Y., Ma Y., Han Z., Zhang Q., Shao Y., Chen J., Kong X. (2008). Identification of a newly isolated avian infectious bronchitis coronavirus variant in China exhibiting affinity for the respiratory tract. Avian Dis..

[66] Xu G., Liu X.Y., Zhao Y., Chen Y., Zhao J., Zhang G.Z. (2016). Characterization and analysis of an infectious bronchitis virus strain isolated from southern China in 2013. Virol. J..

[67] Wang Y.D., Wang Y.L., Zhang Z.C. (1998). Isolation and identification of glandular stomach type IBV (QX IBV) in chickens. Chin. J. Anim. Quar..

[68] Bo Z., Chen S., Zhang C., Guo M., Cao Y., Zhang X., Wu Y. (2022). Pathogenicity evaluation of GVI-1 lineage infectious bronchitis virus and its long-term effects on reproductive system development in SPF hens. Front. Microbiol..

[69] Luo H., Qin J., Chen F., Xie Q., Bi Y., Cao Y., Xue C. (2012). Phylogenetic analysis of the S1 glycoprotein gene of infectious bronchitis viruses isolated in China during 2009–2010. Virus Genes.

[70] Zhao Y., Zhang H., Zhao J., Zhong Q., Jin J.H., Zhang G.Z. (2016). Evolution of infectious bronchitis virus in China over the past two decades. J. Gen. Virol..

[71] Cheng J., Huo C., Zhao J., Liu T., Li X., Yan S., Wang Z., Hu Y., Zhang G. (2018). Pathogenicity differences between QX-like and Mass-type infectious bronchitis viruses. Vet. Microbiol..

[72] Yan S., Liu X., Zhao J., Xu G., Zhao Y., Zhang G. (2017). Analysis of antigenicity and pathogenicity reveals major differences among QX-like infectious bronchitis viruses and other serotypes. Vet. Microbiol..

[73] Xu G., Cheng J., Ma S., Jia W., Yan S., Zhang G. (2018). Pathogenicity differences between a newly emerged TW-like strain and a prevalent QX-like strain of infectious bronchitis virus. Vet. Microbiol..

[74] Kariithi H.M., Volkening J.D., Leyson C.M., Afonso C.L., Christy N., Decanini E.L., Lemiere S., Suarez D.L. (2022). Genome sequence variations of infectious bronchitis virus serotypes from commercial chickens in Mexico. Front. Vet. Sci..

[75] Ghetas A.M. (2021). Infectious bronchitis virus genotypes in the Middle East. Avian Dis..

[76] De Wit J.J., de Wit M.K., Cook J.K.A. (2021). Infectious bronchitis virus types affecting european countries—A review. Avian Dis..

[77] Raj G.D., Jones R.C. (1997). Infectious bronchitis virus: Immunopathogenesis of infection in the chicken. Avian Pathol..

[78] Cheng J., Zhao Y., Hu Y., Zhao J., Xue J., Zhang G. (2021). The furin-S2’ site in avian coronavirus plays a key role in central nervous system damage progression. J. Virol..

[79] Reddy V.R., Trus I., Desmarets L.M., Li Y., Theuns S., Nauwynck H.J. (2016). Productive replication of nephropathogenic infectious bronchitis virus in peripheral blood monocytic cells, a strategy for viral dissemination and kidney infection in chickens. Vet. Res..

[80] Amarasinghe A., Abdul-Cader M.S., Nazir S., De Silva Senapathi U., van der Meer F., Cork S.C., Gomis S., Abdul-Careem M.F. (2017). Infectious bronchitis corona virus establishes productive infection in avian macrophages interfering with selected antimicrobial functions. PLoS ONE.

[81] Barrow A.D., Burgess S.C., Baigent S.J., Howes K., Nair V.K. (2003). Infection of macrophages by a lymphotropic herpesvirus: A new tropism for Marek’s disease virus. J. Gen. Virol..

[82] Chong K.T., Apostolov K. (1982). The pathogenesis of nephritis in chickens induced by infectious bronchitis virus. J. Comp. Pathol..

[83] Tang X., Qi J., Sun L., Zhao J., Zhang G., Zhao Y. (2022). Pathological effect of different avian infectious bronchitis virus strains on the bursa of Fabricius of chickens. Avian Pathol..

[84] Broadfoot D.I., Pomeroy B.S., Smith W.M. (1956). Effects of infectious bronchitis in baby chicks. Poult. Sci..

[85] Crinion R.A.P., Hofstad M.S. (1972). Pathogenicity of four serotypes of avian infectious bronchitis virus for the oviduct of young chickens of various ages. Avian Dis..

[86] Zhong Q., Hu Y.X., Jin J.H., Zhao Y., Zhao J., Zhang G.Z. (2016). Pathogenicity of virulent infectious bronchitis virus isolate YN on hen ovary and oviduct. Vet. Microbiol..

[87] Shao L., Zhao J., Li L., Huang X., Yang H., Cheng J., Liu C., Zhang G. (2020). Pathogenic characteristics of a QX-like infectious bronchitis virus strain SD in chickens exposed at different ages and protective efficacy of combining live homologous and heterologous vaccination. Vet. Res..

[88] Cook J.K., Jackwood M., Jones R.C. (2012). The long view: 40 years of infectious bronchitis research. Avian Pathol..

[89] Jones R.C., Jordan F.T. (1972). Persistence of virus in the tissues and development of the oviduct in the fowl following infection at day old with infectious bronchitis virus. Res. Vet. Sci..

[90] Bisgaard M. (1976). The influence of infectious bronchitis virus on egg production, fertility, hatchability and mortality rate in chickens. Nord. Vet. Med..

[91] Sevoian M., Levine P.P. (1957). Effects of infectious bronchitis on the reproductive tracts, egg production, and egg quality of laying chickens. Avian Dis..

[92] Zhao Y., Xie D., Zhang K., Cheng J., Xu G., Zhang G. (2019). Pathogenicity of a GI-22 genotype infectious bronchitis virus isolated in China and protection against it afforded by GI-19 vaccine. Virus Res..

[93] Cheng J., Zhao Y., Xu G., Zhang K., Jia W., Sun Y., Zhao J., Xue J., Hu Y., Zhang G. (2019). The S2 subunit of QX-type infectious bronchitis coronavirus spike protein is an essential determinant of neurotropism. Viruses.

[94] Hoerr F.J. (2021). The pathology of infectious bronchitis. Avian Dis..

[95] Zhang X., Yan K., Zhang C., Guo M., Chen S., Liao K., Bo Z., Cao Y., Wu Y. (2022). Pathogenicity comparison between QX-type and Mass-type infectious bronchitis virus to different segments of the oviducts in laying phase. Virol. J..

[96] Amarasinghe A., De Silva Senapathi U., Abdul-Cader M.S., Popowich S., Marshall F., Cork S.C., van der Meer F., Gomis S., Abdul-Careem M.F. (2018). Comparative features of infections of two Massachusetts (Mass) infectious bronchitis virus (IBV) variants isolated from Western Canadian layer flocks. BMC Vet. Res..

[97] Li N., Huang C., Chen W., Li Z., Hu G., Li G., Liu P., Hu R., Zhuang Y., Luo J. (2022). Nephropathogenic infectious bronchitis virus mediates kidney injury in chickens via the TLR7/NF-κB signaling Axis. Front. Cell Infect. Microbiol..

[98] Khanh N.P., Tan S.W., Yeap S.K., Lee H.J., Choi K.S., Hair-Bejo M., Bich T.N., Omar A.R. (2018). Comparative pathogenicity of Malaysian QX-like and variant infectious bronchitis virus strains in chickens at different age of exposure to the viruses. J. Comp. Pathol..

[99] Hassan M.S.H., Ali A., Buharideen S.M., Goldsmith D., Coffin C.S., Cork S.C., van der Meer F., Boulianne M., Abdul-Careem M.F. (2021). Pathogenicity of the Canadian Delmarva (DMV/1639) infectious bronchitis virus (IBV) on female reproductive tract of chickens. Viruses.

[100] Boender G.J., Hagenaars T.J., Bouma A., Nodelijk G., Elbers A.R., de Jong M.C., van Boven M. (2007). Risk maps for the spread of highly pathogenic avian influenza in poultry. PLoS Comput. Biol..

[101] Mannelli A., Busani L., Toson M., Bertolini S., Marangon S. (2007). Transmission parameters of highly pathogenic avian influenza (H7N1) among industrial poultry farms in northern Italy in 1999–2000. Prev. Vet. Med..

[102] Singh M., Toribio J.A., Scott A.B., Groves P., Barnes B., Glass K., Moloney B., Black A., Hernandez-Jover M. (2018). Assessing the probability of introduction and spread of avian influenza (AI) virus in commercial Australian poultry operations using an expert opinion elicitation. PLoS ONE.

[103] Franzo G., Tucciarone C.M., Moreno A., Legnardi M., Massi P., Tosi G., Trogu T., Ceruti R., Pesente P., Ortali G. (2020). Phylodynamic analysis and evaluation of the balance between anthropic and environmental factors affecting IBV spreading among Italian poultry farms. Sci. Rep..

[104] Najimudeen S.M., Hassan M.S.H., Cork S.C., Abdul-Careem M.F. (2020). Infectious bronchitis coronavirus infection in chickens: Multiple system disease with immune suppression. Pathogens.

[105] Franzo G., Tucciarone C.M., Blanco A., Nofrarías M., Biarnés M., Cortey M., Majó N., Catelli E., Cecchinato M. (2016). Effect of different vaccination strategies on IBV QX population dynamics and clinical outbreaks. Vaccine.

[106] Moreno A., Franzo G., Massi P., Tosi G., Blanco A., Antilles N., Biarnes M., Majó N., Nofrarías M., Dolz R. (2017). A novel variant of the infectious bronchitis virus resulting from recombination events in Italy and Spain. Avian Pathol..

[107] Sjaak de Wit J.J., Cook J.K.A. (2014). Factors influencing the outcome of infectious bronchitis vaccination and challenge experiments. Avian Pathol..

[108] Alluwaimi A.M., Alshubaith I.H., Al-Ali A.M., Abohelaika S. (2020). The coronaviruses of animals and birds: Their zoonosis, vaccines, and models for SARS-CoV and SARS-CoV2. Front. Vet. Sci..

[109] Ji W., Wang W., Zhao X., Zai J., Li X. (2020). Cross-species transmission of the newly identified coronavirus 2019-nCoV. J. Med. Virol..

[110] Smialek M., Tykalowski B., Dziewulska D., Stenzel T., Koncicki A. (2017). Immunological aspects of the efficiency of protectotype vaccination strategy against chicken infectious bronchitis. BMC Vet Res..

[111] Bande F., Arshad S.S., Bejo M.H., Moeini H., Omar A.R. (2015). Progress and challenges towards the development of vaccines against avian infectious bronchitis. J. Immunol. Res..

[112] Johnson M.A., Pooley C., Ignjatovic J., Tyack S.G. (2003). A recombinant fowl adenovirus expressing the S1 gene of infectious bronchitis virus protects against challenge with infectious bronchitis virus. Vaccine.

[113] Kapczynski D.R., Hilt D.A., Shapiro D., Sellers H.S., Jackwood M.W. (2003). Protection of chickens from infectious bronchitis by in ovo and intramuscular vaccination with a DNA vaccine expressing the S1 glycoprotein. Avian Dis..

[114] Li H., Wang Y., Han Z., Wang Y., Liang S., Jiang L., Hu Y., Kong X., Liu S. (2016). Recombinant duck enteritis viruses expressing major structural proteins of the infectious bronchitis virus provide protection against infectious bronchitis in chickens. Antivir. Res..

[115] Lv L., Li X., Liu G., Li R., Liu Q., Shen H., Wang W., Xue C., Cao Y. (2014). Production and immunogenicity of chimeric virus-like particles containing the spike glycoprotein of infectious bronchitis virus. J. Vet. Sci..

[116] Shi X.M., Zhao Y., Gao H.B., Jing Z., Wang M., Cui H.Y., Tong G.Z., Wang Y.F. (2011). Evaluation of recombinant fowlpox virus expressing infectious bronchitis virus S1 gene and chicken interferon-g gene for immune protection against heterologous strains. Vaccine.

[117] Yang T., Wang H.N., Wang X., Tang J.N., Lu D., Zhang Y.F., Guo Z.C., Li Y.L., Gao R., Kang R.M. (2009). The protective immune response against infectious bronchitis virus induced by multi-epitope based peptide vaccines. Biosci. Biotech. Biochem..

[118] Bijlenga G., Cook J.K., Gelb J., de Wit J.J. (2004). Development and use of the H strain of avian infectious bronchitis virus from the Netherlands as a vaccine: A review. Avian Pathol..

[119] Chhabra R., Forrester A., Lemiere S., Awad F., Chantrey J., Ganapathy K. (2015). Mucosal, cellular, and humoral immune responses induced by different live infectious bronchitis virus vaccination regimes and protection conferred against infectious bronchitis virus Q1 strain. Clin. Vaccine Immunol..

[120] Finney P.M., Box P.G., Holmes H.C. (1990). Studies with a bivalent infectious bronchitis killed virus vaccine. Avian Pathol..

[121] Lee H.J., Youn H.N., Kwon J.S., Lee Y.J., Kim J.H., Lee J.B., Park S.Y., Choi I.S., Song C.S. (2010). Characterization of a novel live attenuated infectious bronchitis virus vaccine candidate derived from a Korean nephropathogenic strain. Vaccine.

